# Comparison of spermatic cord ligation and the vas plexus ligation in canine orchiectomy: A prospective clinical study

**DOI:** 10.1002/vms3.1208

**Published:** 2023-07-19

**Authors:** Mümin Gökhan Şenocak

**Affiliations:** ^1^ Department of Surgery, Faculty of Veterinary Medicine Atatürk University Erzurum Turkey

**Keywords:** canine castration, orchiectomy, self‐tying, spermatic cord ligation, thermal camera, vas deferens pampiniform plexus ligation

## Abstract

**Background:**

Orchiectomy with a vas deferens to pampiniform plexus ligation (VPL) is a novel method, and it is unclear how its short‐term outcomes compare with the results of a conventional method, spermatic cord ligation (SCL).

**Objective:**

To compare the short‐term outcomes of SCL and VPL on inflammation, surgery time, bleeding, pain and surgeon satisfaction during canine open orchiectomy.

**Methods:**

Thirty male crossbred dogs undergoing open orchiectomy were enrolled the study. Dogs were randomly allocated to one of the SCL or VPL groups, with 15 patients in each. In the SCL group, the spermatic cord was ligated using absorbable sutures. The vas deferens, and pampiniform plexus self‐tying were performed in the VPL group. Surgery time, bleeding and surgeon satisfaction scores were recorded. Inflammation at the surgical site was assessed using infrared thermal camera over three days, and pain associated with inflammation was scored on the third day.

**Results:**

On Day 3, the average temperature in the SCL group was significantly higher than that of the VPL group, with a mean difference of 4.63°C (95% CI: 2.34–6.93, *p* < 0.001). Moreover, the surgery time in the VPL group was significantly longer compared to the SCL group, with a mean difference of 1.7 min (95% CI: 0.28–3.11, *p* = 0.021). The bleeding score was also significantly higher in the VPL group (*p* = 0.012). On the other hand, surgeon satisfaction and pain scores were not significantly different between groups.

**Conclusion:**

Both SCL and VPL methods are safe and effective for orchiectomy in dogs. VPL is comparable in efficacy and safety and has the additional benefit of less inflammation.

## Graphical abstract

This study compared orchiectomy with vas deferens to pampiniform plexus ligation (VPL) and conventional spermatic cord ligation (SCL) in 30 male dogs, divided into two groups. Inflammation was higher in the SCL group, whereas the VPL group had longer surgery time and higher bleeding score. However, surgery satisfaction and pain scores were similar between the two methods. Both SCL and VPL are safe and effective for dog orchiectomy. VPL is equally effective and safe, with the added advantage of reduced inflammation.

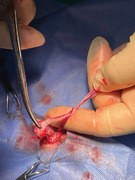

## 1 INTRODUCTION

Canine orchiectomy, also known as an orchidectomy, is a commonly performed surgical procedure in male dogs that involves the removal of the testicles. This procedure is typically performed for medical or behavioural reasons, including preventing unwanted pregnancies, treating testicular tumours and managing hormone‐mediated behaviours (Griffin et al., 2016).

Spermatic cord ligation (SCL), a commonly used method for canine orchiectomy involving clamping and ligating the spermatic cord to prevent blood flow to the testicles, has been widely accepted as safe and effective (Van Goethem, 2017). However, SCL has potential disadvantages, including ligation slippage, the presence of foreign materials in the body, the cost of suture material and the time required for suturing (Gracia‐Calvo et al., 2012). Another drawback of this technique is the risk of post‐operative bleeding, which may result from ligature loosening or inadequate occlusion of blood vessels, leading to complications, such as haematoma formation, wound dehiscence and post‐operative pain (Kongara et al., 2013; Van Goethem, 2017; Yiapanis et al., 2021).

The SCL can cause inflammation and irritation, leading to discomfort and pain for the animal. This can negatively affect the animal's overall welfare and require additional pain management strategies (Miller et al., 2018). Furthermore, SCL can result in a prolonged recovery period and increased surgical time due to the need for the careful dissection and ligation of the cord (Höglund et al., 2014).

Given these disadvantages, there is a growing interest in finding new approaches for canine orchiectomy that can provide similar or improved outcomes while reducing the risk of complications and discomfort for the animal.

This study hypothesized no significant difference between conventional SCL and vas plexus ligation (VPL) on surgeon satisfaction, surgery time, bleeding, inflammation and pain in dogs undergoing open orchiectomy.

## 2 MATERIALS AND METHODS

### 2.1 Animals

This study included 30 healthy male crossbred dogs, aged 1–9 years, weighing 33.4 ± 4.99 kg. The dogs were scheduled to undergo elective orchiectomy as part of a spaying and neutering program at the Atatürk University Veterinary Teaching Hospital.

A physical examination was carried out to assess the animal's health status. The inclusion criteria comprised an American Society of Anaesthesiologists (ASA) physical status score of I, a complete blood count and chemistry panel indicating good health, a body condition score of three to five out of nine and no history of systemic disease. In addition, the animal had to be older than 12 months and younger than ten years of age and show no signs of cardiovascular disease during the clinical examination. Exclusion criteria included the presence of wounds or dermatitis on the scrotum or peri‐scrotal region and ectopic testis. In addition, dogs with rectal temperatures outside the normal range of 37.7–39.1°C are also excluded as part of the presurgical evaluation (Hall & Carter, 2017; Yanmaz et al., 2015).

The study was conducted in a controlled environment maintained at a temperature range of 20–22°C. The animals were housed individually in cages. Prior to anaesthesia induction, the animals were allowed unrestricted access to water, but food was withheld for six hours before the study.

### 2.2 Study design

The experimental design of the research was a randomized unmasked prospective clinical trial.

To determine which group the dogs brought to the hospital would be allocated into, the two sides of a metal coin were labelled SCL and VPL. It was decided that the first 15 dogs brought to the hospital would undergo surgery using the method associated with the side of the coin that remained face up, whereas the later 15 dogs would undergo surgery using the method associated with the side of the coin that remained face down. The personnel performing the coin flipping and bringing the dogs to the hospital were unaware of the working groups. With this method, dogs were divided into SCL and VPL, with 15 dogs in each group.

### 2.3 Anaesthesia and analgesia

Prior to surgery, a single subcutaneous dose of butorphanol tartrate (Butomidor, Richter Pharma) at a rate of 0.4 mg/kg was administered to provide analgesia.

Ketamine (Ketasol 100 mg/mL, Interhas, Richter Pharma AG) and propofol (20 mg/mL, Fresenius Kabi) were combined in a syringe at a ratio of 1/2. To prepare the combination, 5 mL of ketamine and 50 mL of propofol were mixed together. The combined solution, known as ketofol, was administered intravenously through the cephalic vein as a bolus at a rate of 0.2 mL/kg/min. The administration lasted until the relaxation of the jaw was observed, and this initial bolus was recorded. To maintain anaesthesia, additional top‐ups of the same volume as the initial bolus were given. The timing for administering the additional top‐ups was determined through the monitoring of two to three variables: heart rate, mean arterial pressure and respiratory rate. The criterion for administering a top‐up was an increase of more than 20% in these variables from the baseline value. Each top‐up was administered over a duration of 120 s.

### 2.4 Intubation and monitoring

With the loss of jaw tone, the larynx and epiglottis were desensitized with 0.1 mL of lidocaine spray (10%, Vemcaine), and an endotracheal tube was placed into the trachea and fixed to the lower jaw.

The pulse oximeter infrared probe was initially positioned on the ear to gather baseline data and subsequently relocated to the tongue following anaesthesia induction in order to acquire measurements of heart rate and oxygen saturation. Electrocardiogram leads were affixed to the forelimbs and left hindlimb to monitor heart rate and respiratory rate. Additionally, a blood pressure cuff, approximately 40% of the limb circumference in diameter, was promptly positioned proximal to the tarsus on the right pelvic limb to assess indirect systolic, diastolic and mean arterial pressure.

### 2.5 Infrared thermal imaging

In order to monitor the possible occurrence of inflammation after the surgical procedure, measurements were taken of the temperature at the surgery site. The dogs were placed in left lateral recumbency on a surgical table. The peri‐scrotal area was meticulously shaved with a 40‐number trimmer to prevent any injury that could alter the skin temperature. The temperature of the surgical site was then recorded via a thermal infrared camera (IR FlexCam S, Infrared Solutions Inc.) at three distinct points on the site: the estimated pre‐scrotal incision line (IT), the midpoint between the incision line and the scrotum (MT) and the midpoint of the scrotum (ST). The highest temperature of the monitored location was recorded.

Local temperature was monitored using thermal camera at three perisurgical sites for 3 days. Day 1 was used to measure the presurgical temperature, Day 2 for the temperature on the second day after surgery, and Day 3 for the temperature on the third day after surgery.

### 2.6 Surgery

**FIGURE 1:**
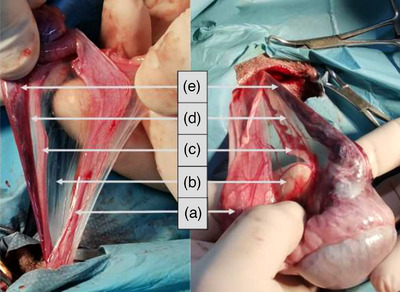
The contents of the spermatic cord following a stab incision made in the vaginal tunic: (a) vaginal tunic comprising visceral and parietal leaves; (b) broad ligament connecting the visceral vaginal tunic to the vas deferens; (c) vas deferens connected to the pampiniform plexus and visceral vaginal tunic; (d) broad ligament connecting the pampiniform plexus and vas deferens; (e) pampiniform plexus.

**FIGURE 2:**
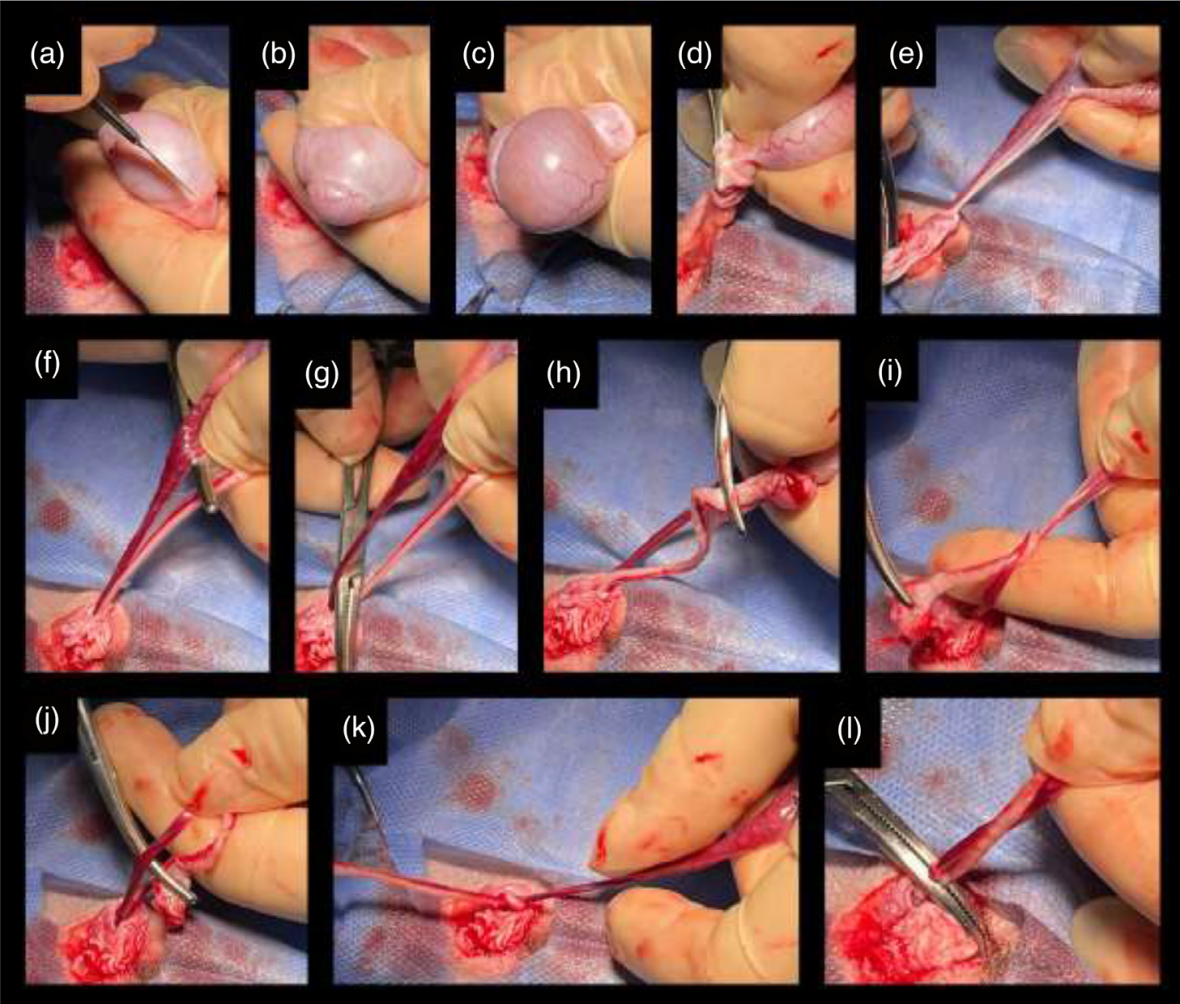
Surgery of the vas deferens plexus pampiniformis ligation (VPL). The testis with epididymis was subsequently released from the mesorchium and mesofuniculum by making a longitudinal stab incision of the parietal tunic (a). The contents were then extruded by compressing the testicle (b and c). The caudal gubernaculum was detached and removed with mosquito forceps (d and e). The broad ligament was bluntly stripped (f and g). The vas deferens were then separated from the epididymis (h) and tied over the pampiniform plexus (i and j) with five square knots (k). The last two knots were squeezed with forceps (l).

The surgical site was prepared in accordance with standard protocols and draped appropriately. A standard pre‐scrotal midline stab incision was made, extending 5 cm cranial to the scrotal edge. One of the testes was then advanced to the incision line and extruded, grasped and pulled out. The epididymis’ peri‐testicular junctions, scrotal fascia and caudal tail ligament were gently stripped using sterile gauze. The testis with epididymis was subsequently released from the mesorchium and mesofuniculum by making a longitudinal stab incision of parietal and visceral vaginal tunics (Figures 1a and 2a). The contents were then extruded by compressing the testicle (Figure 2b,c). The caudal gubernaculum was detached and removed using mosquito forceps (Figure 2d,e). Additionally, the broad ligament (Figure 1b) connecting to the vas deferens (Figure 1c) was bluntly stripped. Subsequently, the surgery proceeded using either SCL or VPL method.

In the VPL approach, an aperture was made by tearing the secondary broad ligament (Figure 1d) located between the vas deferens and pampiniform plexus (Figure 1e), which was further extended to the gubernaculum (Figure 2f,g).

The vas deferens were then separated from the epididymis using mosquito forceps (Figure 2h) and tied over the pampiniform plexus with five square knots (Figures 2i–k). The last two knots were squeezed with Kocher forceps (Figure 2l), and the spermatic cord was excised with the testicle above the knots. Then the forceps was released. For the VPL approach, no suturing material was used; the vas deferens and pampiniform plexus were self‐tied to each other.

In the traditional SCL approach, the spermatic cord was ligated with 3/0 PDS II using three knots: a square knot, a surgeon's knot and a half‐hitch knot. The cord was then excised above the knot.

In both procedures, both testicles were removed through the same incision line, and the subcutaneous tissues and skin were sutured using 3/0 PDS II. Surgery times and complications were documented.

During surgery, bleeding was evaluated by an intern student who was blinded to the study using the Modified Boezaart's scale: 0, no bleeding; 1, slight bleeding with no compression required; 2, slight bleeding with compression required; 3, bleeding with occasional compression required; 4, bleeding with frequent compression required; 5, uncontrollable faster bleeding (Boezaart et al., 1995).

A surgeon who is board‐certified performed both the surgery and assessed the surgeon satisfaction score. The evaluation of surgeon satisfaction was conducted using a five‐point Likert scale with the following descriptions: 1, very poor, there is concern that the knots will be untied after the operation, and the application of the method is deemed impossible to be included in routine practice; 2, poor, there is undecided concern that the knots will be untied after the operation, and the application of the method is not yet determined to be included in routine practice; 3, undecided, there is no definite conclusion regarding the probability of knots becoming untied after the operation, and the suitability of the method for routine practice remains unclear; 4, good, the surgeon is confident that the knots are secure, but it is uncertain whether this method can be easily integrated into routine practice; 5, excellent, the surgeon is highly confident that the knots are secure and that the method can be seamlessly incorporated into routine practice.

Upon the conclusion of the surgery, the dogs were relocated to individual kennels. Over the course of 3 days, the surgeon closely monitored them for any signs of bleeding, hematoma or swelling.

On the third day, the pain assessment was carried out by two intern students who were unaware of the study. The assessment involved touching the surgery site with wet cotton and scoring the pain using a modified numerical rating scale. The scale ranged from 0 to 5, with higher scores indicating a higher intensity of pain: 0, no pain; 1, low pain with just salivation and swallowing; 2, low pain with salivation and swallowing and turning head to the surgery site; 3, mild pain with turning head to the surgery site and putting the tail between legs; 4, mild pain with putting the tail between legs and vocalization present; 5, severe pain with vocalization, and changing body position and trying to escape.

All the images included in this article were originally collected during the course of the study, specifically for educational presentation purposes. These images were gathered with the intention of enhancing the clarity and comprehensibility of the material presented.

### 2.7 Statistical analysis

The average temperature (AT) was calculated as the mean of the temperatures recorded at the IT, MT and ST points. The degree of inflammation at the surgical site was evaluated based on the AT.

The sample size for the study was calculated using MedCalc Software Ltd (v20.013), considering a minimum mean difference of 2.7°C between the groups, as reported by a previous study (Cugmas et al., 2020). It was determined that a minimum of four animals per group would be required to conduct the study with an alpha (Type I error) level of 0.05 and a power (Type II error) of 0.8. Although a minimum of four animals per group is required for the study to be conducted, data from all animals scheduled for castration was included in the study to enhance its power.

The normality of the data was assessed using the Kolmogorov–Smirnov test. Levene's test was performed to assess the homogeneity of variances. An independent sample *t*‐test was used to compare the two treatment groups’ surgery time, AT and rectal temperature.

Repeated measures ANOVA was utilized to examine the differences in temperature over the 3 days of monitoring. The surgeon satisfaction, bleeding and pain scores were compared using the Mann–Whitney *U* test between the two treatment groups.

The parametric data is presented as mean ± standard deviation. The nonparametric data is given as the median rank and range. Mean differences between combinations are presented as mean differences (95% CI lower bound–upper bound). A level of alpha (Type I error) 0.05 is considered statistically significant.

Statistical analyses were performed using SPSS software (Version 22, IBM Corp.).

## 3 RESULTS

Surgical interventions were completed without complications, such as swelling of the scrotum, oedema, haematoma or abscess around the incision line within the study period. None of the dogs were excluded from the study due to the presence of wounds or dermatitis on the scrotum, peri‐scrotal region or ectopic testis.

The average rectal temperature was 38.72 ± 0.31°C without significant differences between SCL and VPL groups (*p* = 0.315).

In the SCL group, a statistically significant difference was found between the ATs on Days 1 and 2, with a mean difference of 3.88°C (95% CI: 1.71–6.05, *p* < 0.001) and between Days 1 and 3, with a mean difference of 3.85°C (95% CI: 2.34–5.35, *p* < 0.001). However, the difference in AT between Days 2 and 3 was not significant, although Day 3 was not colder than Day 2, with a mean difference of −0.04°C (95% CI: −2.04 to 1.97, *p* > 0.05).

In the VPL group, there were significant differences in ATs between Days 1 and 2 with a mean difference of 6.24°C (95% CI: 3.46–9.01, *p* < 0.001), and between Days 1 and 3 with a mean difference of 8.42°C (95% CI: 5.45–11.49, *p* < 0.001). The AT between Days 2 and 3 was insignificant, but Day 3 was cooler than Day 2 with a mean difference of 2.24°C (95% CI: −1.57 to 6.04, *p* = 0.385).

It was observed that there were no significant differences in the ATs between the VPL and SCL groups on Days 1 and 2. However, on Day 3, the AT in the SCL group was significantly higher than that of the VPL group, with a mean difference of 4.63°C (95% CI: 2.34–6.93, *p* < 0.001).

Moreover, the surgery time in the VPL group was significantly longer compared to the SCL group, with a mean difference of 1.7 min (95% CI: 0.28–3.11). Additionally, the bleeding score was also found to be significantly higher in the VPL group (*p* = 0.012). On the other hand, surgeon satisfaction and pain scores were not significantly different between groups (Table 1).

**TABLE 1:** Comparison of the short‐term outcomes of spermatic cord ligation (SCL) and VPL, on inflammation, surgery time, bleeding, pain and surgeon satisfaction during canine open orchiectomy.

Variable		SCL group	VPL group	*p*‐Value^a^
*n*		15	15	
Age (years)		5.92 ± 3.22	5.38 ± 2.63	0.645
Weight (kg)		33.46 ± 5.87	33.69 ± 4.05	0.908
Rectal temperature (°C)		38.66 ± 0.35	38.78 ± 0.26	0.315
Body condition score (1–9)		4 (3–5)	4 (3–5)	0.340
Surgery time (minutes)		13.23 ± 1.54	14.92 ± 1.93	0.021
Satisfaction score (1–5)		4 (4–5)	5 (4–5)	0.243
Bleeding score (1–5)		1 (1–2)	2 (1–2)	0.012
Pain score (1–5)		2 (1–3)	2 (1–3)	0.054
Average temperature (°C)	Day 1	36.06 ± 0.98	36.05 ± 0.70	0.970
	Day 2	32.18 ± 2.47	29.81 ± 3.59	0.062
	Day 3	32.21 ± 1.69	27.58 ± 3.64	<0.001

Abbreviation: VPL, vas deferens to the pampiniform plexus ligation.

^a^ *p*‐Values were determined by independent samples *t*‐test for surgery time, average and rectal temperatures. Scores were compared with Mann–Whitney *U* test between groups.

## 4 DISCUSSION

The current study has ascertained that both conventional SCL and VPL methods are satisfactory for surgeon when performing canine orchiectomy. SCL exhibits a shorter duration of surgery and less bleeding, whereas VPL causes less inflammation at the surgical site. Therefore, the null hypothesis has been rejected for inflammation.

In the present study, the detection of inflammation was accomplished using a thermal imaging method. The temperature of the skin can indicate the presence of inflammation in underlying tissues or abnormalities in blood flow. Thermal imaging devices detect the infrared energy emitted by the skin, making it easier to detect temperature changes that are associated with inflammation (Ring & Ammer, 2012). Thermal imaging presents itself as a potential alternative for inflammation detection. According to one study, mastitis detection can be accomplished through thermal cameras (Hovinen et al., 2008). Another study suggests that thermal imaging may effectively diagnose inflammatory ocular conditions (Kawali, 2013). Additionally, a study conducted on horses found that inflammatory conditions can be detected using thermal imaging (Okur et al., 2023).

Thermal imaging analysis indicated that the mean AT difference between the first and third days was higher in the SCL group (3.85 ± 0.71) compared to the VPL group (8.47 ± 2.94). This higher temperature observed in the SCL group suggests a greater degree of inflammation, which could be attributed to the suture material used in the SCL group acting as a foreign body and triggering an inflammatory response. This response has been observed in several studies, which have demonstrated that using absorbable suture materials can lead to the development of an inflammatory response due to the generation of an external antigen (Farrar & Binns, 1997; LaBagnara, 1995; Titley‐Diaz & De Cicco, 2022). The ligation of the spermatic cord in the VPL method was achieved without sutures, which led to a lower inflammatory response at the surgical site compared to the SCL group. Furthermore, the absence of sutures in the VPL method reduced the cost of the suture material. This cost‐saving measure is essential in castration surgery, as it is a well‐known economic concern, especially in animal shelters that deal with large numbers of dogs. By reducing expenses, such shelters can prevent significant losses in the long run (Silva et al., 2020).

Although the most common method is SCL during canine orchiectomy, this method has limitations. SCL may slip and cause foreign material effects, expense and time to repair (Gracia‐Calvo et al., 2012; Van Goethem, 2017). SCL may cause post‐operative haemorrhage. Haematoma, wound dehiscence and post‐operative discomfort might result from slack ligatures or inadequate blood vessel occlusion (Kongara et al., 2013; Van Goethem, 2017; Yiapanis et al., 2021). The present study showed that SCL was a satisfactory method for performing canine orchiectomy. However, the VPL method was also found to be comparably effective, and the surgeon reported similar satisfaction levels compared to SCL. Notably, the VPL method involved self‐tying ligations. It did not require suture material for tying the spermatic cord, which may provide advantages over SCL by reducing the risk of slippage of the knots. Although both methods were performed by the same surgeon, the VPL method took, on average, 1.69 ± 0.39 min longer than the SCL method. This difference in duration could be attributed to the surgeon's initial lack of experience in performing the new method.

Despite the availability of various methods for performing sutureless orchiectomy in kittens, juvenile dogs and cats (Miller et al., 2018), there is currently no alternative sutureless technique suitable for adult dogs with large spermatic cords. Unlike complete autoligation of the spermatic cord, the VPL method involves making four to five ligations from the pampiniform plexus to the vas deferens. By excluding the internal spermatic fascia, external spermatic fascia and Cremaster muscle from the knots, the resulting knots are small and safe. This key distinction sets the VPL method apart from the autoligation technique and enables its utilization even in adult dogs with large spermatic cords.

It is important to note several limitations of this study. First, the total oxidative stress of both methods and cortisol levels were not compared as the study was conducted as a part of the undergraduate education material and additional budget could not be used. Second, although both methods are generally safe, there is a potential predisposition for acquired arteriovenous fistula as a complication, and long‐term outcomes need to be evaluated and compared to fully assess the safety of these methods. Thus, further research is necessary to fully explore these methods’ potential risks and benefits in clinical practice.

## 5 CONCLUSIONS

Both SCL and VPL are effective and safe for performing orchiectomy in dogs. Although SCL is widely accepted and commonly used, VPL has been found to be comparable in safety and efficacy, with the added benefit of less inflammation and cost. Furthermore, in the VPL method, there is no risk of suture material slippage, thereby helping to prevent potential complications and reduce the necessity for additional surgical interventions. Ultimately, the choice between SCL and VPL will depend on the individual needs and circumstances of the animal, as well as the preferences and expertise of the veterinary surgeon. However, the evidence suggests that VPL is a viable alternative to SCL for performing orchiectomy in dogs, with potential benefits for both the animal and the owner.

## AUTHOR CONTRIBUTIONS

*Conceptualization; data curation; formal analysis; investigation; methodology; project administration*: Mümin Gökhan Şenocak.

## CONFLICT OF INTEREST STATEMENT

The author does not have any potential conflict of interest to declare.

## FUNDING INFORMATION

None.

### PEER REVIEW

The peer review history for this article is available at https://publons.com/publon/10.1002/vms3.1208.

## ETHICS STATEMENT

The study was conducted with the permission of the Atatürk University Local Boards of Ethics Committee (Session no: 2022/7, Decision No: 129), and the study was conducted in adherence to the directives prescribed by the National Institutes of Health Guide for the Care and Use of Laboratory Animals.

## ANIMAL WELFARE

The authors confirm that they have adhered to ARRIVE (Animal Research: Reporting of In Vivo Experiments) Guidelines to protect animals used for scientific purposes.

## Data Availability

The data that support the findings of this study is available from the corresponding author upon reasonable request.
